# Does the Type of Temporary Housing Make a Difference in Social Participation and Health for Evacuees of the Great East Japan Earthquake and Tsunami? A Cross-Sectional Study

**DOI:** 10.2188/jea.JE20180080

**Published:** 2019-10-05

**Authors:** Taro Kusama, Jun Aida, Kemmyo Sugiyama, Yusuke Matsuyama, Shihoko Koyama, Yukihiro Sato, Takafumi Yamamoto, Ayaka Igarashi, Toru Tsuboya, Ken Osaka

**Affiliations:** 1 Department of International and Community Oral Health, Tohoku University Graduate School of Dentistry, Miyagi, Japan; 2 Miyagi Prefectural Government Office, Miyagi, Japan; 3 Department of Global Health Promotion, Graduate School of Medical and Dental Sciences, Tokyo Medical and Dental University (TMDU), Tokyo, Japan; 4 Japan Society for the Promotion of Science, Tokyo, Japan; 5 Division of Community Oral Health Science, Department of Community Medical Supports, Tohoku Medical Megabank Organization, Miyagi, Japan; 6 Division of Public Health and Epidemiology, Department of Social Medicine, Asahikawa Medical University, Hokkaido, Japan; 7 Disaster Medical Science Division, Disaster Related Oral Health, International Research Institute of Disaster Science, Miyagi, Japan

**Keywords:** evacuation, social participation, health condition, the Great East Japan Earthquake population attributable fraction

## Abstract

**Background:**

Although the majority of survivors of the huge Great East Japan Earthquake and Tsunami evacuated to two types of temporary housings, prefabricated housing and rented housing, health effects of these different environments were unclear. We examined whether prevalent social participation in prefabricated housing brought larger health benefits than in rented housing using the largest health survey data of the disaster survivors.

**Methods:**

This cross-sectional study used a 2012 survey by the Miyagi Prefectural Government, in which almost all of evacuees were targeted (response rate: 61.6%). Self-rated health (SRH) and psychological distress measured via K6 score were the dependent variables, and social participation was the independent variable. Odds ratios of the social participation on health variables were estimated using logistic regression models. To assess the contribution of social participation, the population attributable fraction (PAF) was estimated.

**Results:**

The participants lived in prefabricated and rented housing numbered 19,726 and 28,270, respectively. Participants in prefabricated housing had poorer SRH and K6 than those in rented housing. The proportions of participants engaging in social participation of prefabricated and rented housing were 38.2% and 15.4%, respectively. The absence of social participation was significantly associated with poor SRH and K6 among participants in both housing types. The PAFs of social participation with good SRH were 39.5% in prefabricated housing and 14.4% in rented housing. For K6, the PAFs were 47.1% and 19.5% in prefabricated and rented housing, respectively.

**Conclusion:**

Compared to the residents in rented housing, residents in prefabricated housing had more frequent opportunities for social participation, which was associated with larger health benefits.

## INTRODUCTION

When large-scale disasters destroy living environments, victims are forced to move to temporary accommodations.^1^ Previous studies have reported that forced migration exacerbated evacuees’ physical and mental health in both the short and long term.^2^^–^^4^ After the Great East Japan Earthquake and Tsunami, evacuees’ health conditions worsened owing to the change in living environment, even apart from health problems caused directly by the disaster.^5^^–^^9^ In the Americas, after Hurricane Katrina, a rise in the prevalence of several diseases, including heart disease, cardiovascular disease, hypertension, and sleep problems, was observed.^10^

On the other hand, social participation is known to help increase and maintain individuals’ social networks,^11^ which are important sources of psychological and emotional support. Previous studies of various populations have reported on the benefits of social participation for health conditions, including mental health.^12^^–^^16^ It was also reported that the contribution of social participation for good health is beneficial among disaster survivors.^17^

The Great East Japan Earthquake in 2011 resulted in 19,533 human deaths and the complete or partial destruction of 401,928 buildings.^18^ In addition, many surviving victims were forced to migrate and settle in temporary housing. They were provided two types of temporary housing.^19^^,^^20^ One is prefabricated temporary housing (prefabricated housing), where many evacuees live close together. Prefabricated houses are built with thin panels, and the air conditioning system is insufficient for living there for long periods. After the Great East Japan Earthquake, the government needed to build a large number of temporary houses. Therefore, the structure of the temporary houses was simple, and the minimum equipment was installed. In Japan, the temperature and humidity are very high in the summer, and there is snowfall and cold temperatures in the winter. As such, the physical living environment of prefabricated housing could deteriorate evacuee health. Although this situation is accompanied by challenges, such as noise and difficulty controlling the temperature and humidity,^21^ there are some benefits. For example, residents of prefabricated housing have better access to internal and external social supports and opportunities for social participation. Local municipalities and volunteers held events, like movies or concerts, and also facilitated activities, like tea parties and exercise programs. The other type of housing for evacuees is private rented housing (rented housing), which evacuees rent with financial support from the local government. They settled in a community where non-victimized people were dominant. The housing was often scattered throughout a community. Therefore, it was sometimes difficult for support opportunities from the local governments or volunteers to reach to the residents of rented houses. Some support events, such as holding cafeterias for the victims, were carried out in prefabricated housing colonies where victims were clustered. For volunteers from different areas, transportation to prefabricated housing provided an easy way to reach the victims. Therefore, they may find it challenging to find opportunities for social participation.

To date, few studies have investigated the implications of these differences in the social environment of prefabricated versus rented housing for evacuees, especially targeting wide areas. Miyagi Prefecture was the prefecture with the greatest disaster damage at the Great East Japan Earthquake, and the prefecture conducted a health survey for all survivors. We hypothesized that different types of housing can influence the association between evacuees’ social participation and their health. Thus, we examined whether prevalent social participation among the residents in prefabricated temporary housing explained the difference of health between the evacuees using data from the largest health survey of the disaster survivors, which was conducted by the Miyagi Prefectural Government.

## METHODS

### Setting and data source

This research uses data from a survey by the Miyagi Prefectural Government. In 2012, the Miyagi Prefectural Government conducted a comprehensive survey of evacuees living in the Miyagi Prefecture, which was severely damaged by the tsunami.^17^ The self-reported questionnaire survey examined personal characteristics and general and mental health conditions among evacuees aged 18 years or older. It was distributed to evacuee families by the staff of local municipality governments and returned via post.

### Participants

The participants included evacuees living in prefabricated housing and those living in rented housing. The evacuees in prefabricated housing were surveyed from September to December 2012. Among 15,979 families, 9,366 participated in the survey (response rate: 58.6%). The evacuees in rented housing were surveyed from December 2012 to March 2013. Among 22,172 families, 14,124 participated (response rate: 63.7%).

### Dependent variables

We applied two health measurements for the dependent variables: self-rated health (SRH)^22^ and psychological distress measured using the Kessler Psychological Distress Scale (K6).^23^ SRH is a simple, single-item question about participants’ health condition. It is considered a valid measurement of health in epidemiological surveys. A previous study reported a strong relationship between low SRH and an increased risk of mortality.^22^ The participants were asked, “What is your physical health condition today?”, and the answer categories were “Very good”, “Good”, “Not so good”, and “Bad”. We dichotomized the responses as follows: “Very good” or “Good” were considered to indicate good SRH, and “Not so good” or “Bad” indicated poor SRH.

K6 was originally developed to screen for non-specific psychological distress in serious mental health research, and its credibility and availability have been documented in previous studies.^23^ We used the Japanese version of the K6 questionnaire, which has been validated.^24^ K6 consists of a six-item battery asking how frequently respondents have experienced symptoms of psychological distress in the past 30 days. The responses range from 0 “none of the time” to 4 “all of the time,” with a total score range of 0 to 24. Following previous studies,^25^^–^^27^ we dichotomized the total score into categories of 13 or over (having severe psychological distress) and 12 or under (not having severe psychological distress).

### Independent variables and covariates

Social participation was used as the independent variable. It was measured through the question “Do you participate in any local events?”. If the answer was “Yes”, we regarded it as an indication of social participation.

Sex, age, occupation, comorbidity, number of family members, and type of temporary housing were the covariates. We categorized age into seven ranges: “18 to 29”, “30 to 39”, “40 to 49”, “50 to 59”, “60 to 69”, “70 to 79”, and “80 or over”. As a proxy of socioeconomic status, occupation was used. We categorized employment status into four categories: “Company employee”, “Self-employed business owner”, “Other (including students and part-time workers)”, and “Not working”. Those who reported as having a disease with medical treatment were categorized as having comorbidity. Family size (defined as the number of family members living in the household, including the respondent) was also considered. We categorized the responses into five categories: “1”, “2”, “3”, “4”, and “5 or over”. We also adjusted the types of temporary housings where evacuees had relocated, and we included social support in the multivariate model to estimate the mediation between social participation and health condition. The question about social support was: “Do you have someone who listens to your concerns?”, and a response of “Yes” was considered to indicate the existence of social support.

### Statistical analysis

We calculated the adjusted odds ratios (aORs) and 95% confidence intervals (CIs) of social participation for poor SRH and K6 scores of 13 and over through multivariate logistic regression analyses, and we also stratified the data by housing types and calculated the aORs and 95% CIs. We built three models. First, a univariate model, including social participation or type of temporary housing, was built, which was not adjusted for any covariates (model 1). Then, all covariates were adjusted (model 2); and, finally, social support as a mediator was included in the model (model 3). To estimate the contribution of social participation to health, we also calculated the population attributable fraction (PAF). PAF is widely used to measure the health impacts of exposure in a particular population. PAF is calculated according to the prevalence of exposure and the risk of exposure on health outcomes, so the comprehensive impact of exposure on health among a particular population can be considered.^28^ PAF is usually used to estimate the disease or mortality proportion that was prevented or reduced when an exposure risk factor was converted to a non-exposed situation. In this study, we calculated the PAF of social participation on good health conditions to evaluate the difference of the contribution of social participation by housing type. For this purpose, we reversed the order of outcomes on health conditions (coded good health as 1 and poor health as 0), re-calculated the OR using multivariate regression analysis, and calculated the PAF using the OR. We used multiple imputation (MI) to reduce bias arising from the influence of missing information.^29^ The original database was created to be imputed, and multivariate logistic analysis was carried out. We used Stata MP version 14.1 (Stata Corp., College Station, TX, USA) for the statistical analysis.

### Ethical issues

This study used the data obtained from the Miyagi Prefectural Government before the planning of our study. We obtained an anonymized data set from the prefectural office. Ethical approval for secondary data analysis was obtained from the Ethics Committee of Tohoku University Graduate School of Dentistry.

## RESULTS

Table 1 shows the characteristics of the evacuees in each group before MI. The participants in prefabricated housing were more likely to engage in social participation than those in rented housing (prefabricated housing, 38.2%, *N* = 7,534; rented housing, 15.4%, *N* = 4,345). There were differences in the characteristics of participants in the two types of accommodations: people in prefabricated housing were older than their counterparts in rented housing. Mean age and standard deviation (SD) of participants was 57.6 (SD, 18.0) years old among participants in prefabricated housing and 51.7 (SD, 18.4) years old among participants among in rented housing. The gender distribution was almost the same between the two housing groups: the proportion of males was 46.7% among participants in prefabricated housing and 46.2% among participants in rented housing.

**Table 1.** Characteristics of participants in prefabricated housing and rented housing^a^

Characteristics	All participants (*N* = 47,996)		Prefabricated temporary housing (*N* = 19,726)		Private rented housing (*N* = 28,270)	
		
	Number	%	Number	%	Number	%
Self-rated health						
Good	36,885	76.9	14,683	74.4	22,202	78.5
Poor	9,406	19.6	4,021	20.4	5,385	19.1
Mental health condition						
Good	34,323	71.5	12,895	65.4	21,428	75.8
Poor	3,138	6.5	1,320	6.7	1,818	6.4
Social participation						
Yes	11,879	24.8	7,534	38.2	4,345	15.4
No	31,733	66.1	9,974	50.6	21,759	77.0
Age, years						
18–29	5,566	11.6	1,653	8.4	3,913	13.8
30–39	6,669	13.9	1,970	10.0	4,699	16.6
40–49	6,782	14.1	2,505	12.7	4,277	15.1
50–59	7,784	16.2	3,112	15.8	4,672	16.5
60–69	9,487	19.8	4,173	21.2	5,314	18.8
70–79	7,158	14.9	3,711	28.8	3,447	12.2
≥80	3,901	8.1	1,953	9.9	1,948	6.9
Sex						
Male	22,276	46.4	9,220	46.7	13,056	46.2
Female	25,354	52.8	10,140	51.4	15,214	53.8
Occupation						
Company employee	12,608	26.3	3,909	19.8	8,699	30.8
Self-employed business owner	8,337	17.4	2,277	11.5	6,060	21.4
The others (including students and part time job)	11,294	25.5	5,675	28.8	5,619	19.9
Unemployment	14,193	29.6	6,809	34.5	7,384	26.1
Comorbidity						
No	22,234	46.3	7,677	38.9	14,557	51.5
Yes	23,014	48.0	10,547	53.5	12,467	44.1
No. of household member						
1	5,823	12.1	2,713	13.8	3,110	11.0
2	14,413	30.0	6,543	33.2	7,870	27.8
3	12,006	25.0	4,691	23.8	7,315	25.9
4	8,390	17.5	2,975	15.1	5,415	19.2
≥5	6,993	14.6	2,804	14.2	4,189	14.8
Social support						
Yes	34,622	72.1	13,333	67.6	21,289	75.3
No	7,609	15.9	3,432	17.4	4,177	14.8

^a^Omited missing variable.

Table 2 shows the health conditions of participants with or without social participation. Among participants without social participation, the percentage of those in poor SRH was 24.1% in the prefabricated housing group and 20.5% in the rented housing group, and the percentage of those whose K6 scores were ≥13 was 11.1% in the prefabricated housing group and 8.6% in the rented housing group.

**Table 2.** Health conditions of participants with/without social participation^a^

*N* (%)	All participants		Prefabricated temporary housing		Private rented housing	
		
	Social participation		Social participation		Social participation	
	Yes	No	Yes	No	Yes	No
Self-rated health						
good	9,628 (83.5)	24,378 (78.4)	5,952 (82.0)	7,382 (75.9)	3,676 (86.0)	16,996 (79.5)
poor	1,908 (16.5)	6,734 (21.6)	1,311 (18.0)	2,347 (24.1)	597 (14.0)	4,387 (20.5)
Mental health condition						
good	8,527 (94.2)	23,924 (90.7)	5,115 (93.2)	6,984 (88.9)	3,412 (95.8)	16,940 (91.4)
poor	522 (5.8)	2,457 (9.3)	374 (6.8)	870 (11.1)	148 (4.2)	1,587 (8.6)

^a^Excluded missing variable.

Table 3 shows the association between poor SRH and social participation based on logistic regression analysis with MI, in which missing variables of 15,588 participants among all 47,996 participants were imputed. In model 2, among all participants, the absence of social participation was associated with poor SRH (aOR 1.89; 95% CI, 1.78–2.01). Type of temporary housing was significantly associated with poor SRH. The model stratified by housing types also showed the same association among prefabricated housing (aOR 1.92; 95% CI, 1.77–2.09) and rented housing (aOR 1.87; 95% CI, 1.69–2.05). In model 3, the aOR of the absence of social participation did not decrease largely after adjustment for social support.

**Table 3.** The association between social participation and poor self-rated health

	All participants (*N* = 47,996)						Prefabricated temporary housing (*N* = 19,726)						Private rented housing (*N* = 28,270)					
		
	Model 1^a^		Model 2^b^		Model 3^c^		Model 1^a^		Model 2^b^		Model 3^c^		Model 1^a^		Model 2^b^		Model 3^c^	
	OR (95% CI)	*P* value	aOR (95% CI)	*P* value	aOR (95% CI)	*P* value	aOR (95% CI)	*P* value	aOR (95% CI)	*P* value	aOR (95% CI)	*P* value	OR (95% CI)	*P* value	aOR (95% CI)	*P* value	aOR (95% CI)	*P* value
Social participation																		
Yes	Ref.		Ref.		Ref.		Ref.		Ref.		Ref.		Ref.		Ref.		Ref.	
No	1.39 (1.32–1.47)	<0.001	1.89 (1.78–2.01)	<0.001	1.72 (1.62–1.83)	<0.001	1.45 (1.35–1.57)	<0.001	1.92 (1.77–2.09)	<0.001	1.77 (1.63–1.93)	<0.001	1.57 (1.43–1.72)	<0.001	1.87 (1.69–2.05)	<0.001	1.67 (1.51–1.84)	<0.001

Type of temporary housing																		
Private rented accommodation	Ref.		Ref.		Ref.													
Prefabricated temporary housing	1.13 (1.08–1.19)	<0.001	1.08 (1.02–1.13)	0.006	1.02 (0.97–1.08)	0.38												

aOR, adjusted odds ratio; CI, confidence interval; OR, odds ratio.

^a^Univariate model.

^b^Model 2 included both social participation and housing type simultaneously with adjustment for age, sex, occupation, comorbidity, number of household member.

^c^Social support was added to the model 2.

Table 4 shows the association between a K6 score ≥13 and social participation based on logistic regression analysis with MI. In model 2, among all participants, the absence of social participation was associated with a K6 score ≥13 (aOR 2.15; 95% CI, 1.92–2.40). Having relocated to prefabricated housing was associated with a K6 score ≥13 compared to having relocated to rented housing (aOR 1.28; 95% CI, 1.18–1.38). The model stratified by housing types also showed the same association among prefabricated housing (aOR 2.15; 95% CI, 1.88–2.45) and rented housing (aOR 2.21; 95% CI, 1.86–2.64). In model 3, aOR of the absence of social participation did not decrease largely after adjustment for social support.

**Table 4.** The association between social participation and poor mental health

	All participants (*N* = 47,996)						Prefabricated temporary housing (*N* = 19,726)						Private rented housing (*N* = 28,270)					
		
	Model 1^a^		Model 2^b^		Model 3^c^		Model 1^a^		Model 2^b^		Model 3^c^		Model 1^a^		Model 2^b^		Model 3^c^	
	OR (95% CI)	*P* value	aOR (95% CI)	*P* value	aOR (95% CI)	*P* value	OR (95% CI)	*P* value	aOR (95% CI)	*P* value	aOR (95% CI)	*P* value	OR (95% CI)	*P* value	aOR (95% CI)	*P* value	aOR (95% CI)	*P* value
Social participation																		
Yes	Ref.		Ref.		Ref.		Ref.		Ref.		Ref.		Ref.		Ref.		Ref.	
No	1.67 (1.51–1.84)	<0.001	2.15 (1.92–2.40)	<0.001	1.83 (1.63–2.06)	<0.001	1.76 (1.55–2.00)	<0.001	2.15 (1.88–2.45)	<0.001	1.86 (1.62–2.13)	<0.001	2.10 (1.78–2.49)	<0.001	2.21 (1.86–2.64)	<0.001	1.83 (1.53–2.19)	<0.001

Type of temporary housing																		
Private rented accommodation	Ref.		Ref.		Ref.													
Prefabricated temporary housing	1.22 (1.14–1.31)	<0.001	1.28 (1.18–1.38)	<0.001	1.18 (1.09–1.28)	<0.001												

aOR, adjusted odds ratio; CI, confidence interval; OR, odds ratio.

^a^Univariate model.

^b^Model 2 included both social participation and housing type simultaneously with adjustment for age, sex, occupation, comorbidity, number of household member.

^c^Social support was added to the model 2.

**Figure 1.**
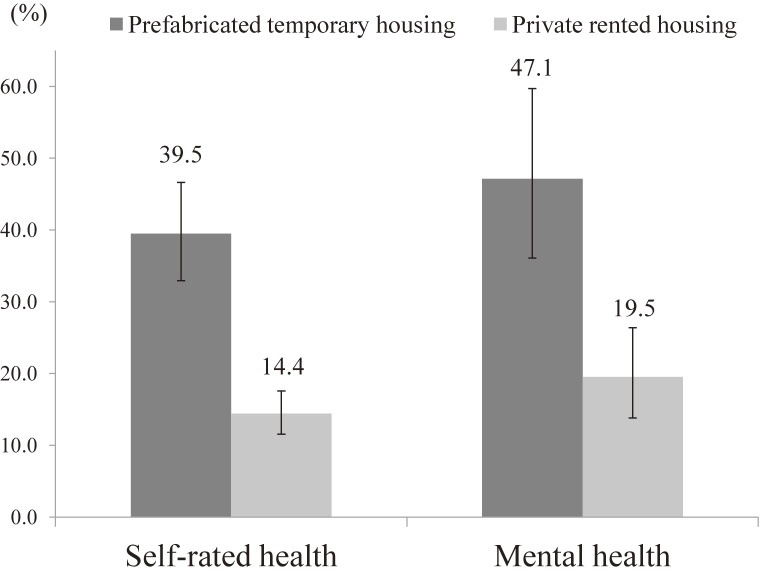
Population attributable fractions (PAFs) of social participation for good self-rated health and good mental health. Population attributable fractions present the contributions by social participation for good self-rated health and good mental health. Overall, the PAFs of social participation differed and were higher in the prefabricated housing group.

The PAFs of the contributions of social participation to good SRH and K6 score ≤12 are shown in Figure 1. Overall, the PAFs of social participation differed and were higher in the prefabricated housing group. For SRH, the PAFs for those whose health conditions were maintained by social participation were 39.5% (95% CI, 32.9–46.6%) in the prefabricated housing group and 14.4% (95% CI, 11.6–17.6%) in the rented housing group. For the K6 score ≤12, they were 47.1% (95% CI, 36.1–59.7%) in the prefabricated housing group and 19.5% (95% CI, 13.8–26.4%) in the rented housing group.

## DISCUSSION

Using data from the largest health survey of the disaster survivors, this study revealed that, compared to the residents of rented housing, the residents of prefabricated housing have more frequent opportunities for social participation, which was associated with a larger health benefit. This is presumably because, in contrast to rented housing, prefabricated housing units are built in the same areas, allowing government workers and volunteers easy access to evacuees to offer them opportunities for social participation.

Previous studies have shown that social participation improves participants’ health conditions.^14^^,^^30^ The present study shows the benefit of social participation for evacuees after a disaster. Similar to the present study, previous disaster-related studies reported a relationship between social factors and health for disaster survivors. Moving away from disaster affected areas destroys evacuees’ social networks, which exacerbates their health.^31^^–^^33^ Social capital, which is brought about by social networks and social participation,^34^ has also been reported to help maintain victims’ health conditions in the post-disaster reconstruction phase.^35^ In addition, social capital contributes to the reconstruction of the community.^36^ Therefore, enhancing social capital in the evacuees’ community could improve their health. In fact, it was reported that interventions to facilitate social participation in local events by evacuees in prefabricated housing improved their health.^37^ As examples of local events for which there were social participation opportunities, previous studies reported that events like movies, concerts, tea parties, and exercise programs were held for the residents living in temporary housing after the Great East Japan Earthquake.^19^^,^^38^

In this study, we included social support in the multivariate model. As a result, the role of social support as a mediator between social participation and health condition seemed to be smaller. This result may be because of the limitations of the questionnaire: the question we used to assess social support focused only on emotional social support, and instrumental and informational support may not have been fully assessed using this question. Therefore, the mediation effect of social support was smaller than we expected. Social participation also provides instrumental and informational support,^39^ so it is possible that, if we could ask about instrumental and informational support, the mediation effects of social support would increase. Additionally, most of the participants in this study had social support. The proportion of those who had social support was over 70%. Meanwhile, that of those who had social participation was around 25%; therefore, from these figures it is apparent that most people gained emotional social support regardless of their social participation. In this situation, the mediation effect might be underestimated.

There are several strengths and limitations to this research. Its strength lies in being a large-scale study that includes 47,000 evacuees living in Miyagi Prefecture, in both prefabricated housing and rented housing. By contrast, previous studies that have examined the relationship of social factors and health condition conducted surveys in only one to several municipalities^40^^–^^43^ or targeted only prefabricated housing.^17^ The survey targeting residents living in rented housing was difficult to conduct because these individuals are scattered throughout the community. However, this study used a survey conducted by the prefectural government, so we were able to include a large number of participants who lived in rented housing. However, one limitation was that the response rate was moderate, which decreases the external validity of the present study results. Given this lower response rate, it is possible that residents who had poorer health or less social participation tended to not answer the questionnaire. Based on the present logistic regression model, we checked the linearity of the estimated association between social participation and health and found it was almost linear (not shown). Therefore, if the response rate had been higher, the difference in the estimated association might have been similar. Additionally, because this was a cross-sectional study, PAF was calculated by comparing the prevalence of the outcome according to housing type.^28^ Incidence of poor health was not used, and prevalence of poor health status was applied to the calculation. Because it is possible the poor health of some participants occurred before the disaster or relocation, estimated PAF may have been inaccurate, or over- or under-estimated. Although PAF has often been estimated in cross-sectional studies,^28^ an estimation from cohort studies is required. This study did not use variables on the damage degree from disaster in each individual. However, we do not believe this limitation affected our results much because most participants’ homes were destroyed in the earthquake and tsunami. It is possible that there was some kind of contextual effect related to social participation in relation to the prefabricated housing.^17^ However, because the main objective of the present study was to reveal the different contributions of social participation on health in two types of housing, we did not distinguish contextual from compositional effects. Therefore, our estimated results concerning social participation were considered to include contextual effects relating to social participation, especially in prefabricated housing communities. Thus, the present results suggest differences in the total effects of social participation between prefabricated and rented housing. We used a single-item question to assess social participation because we could not obtain the intensity or frequency of social participation. Although our result was consistent with previous studies,^17^ we could not determine the frequency threshold of social participation. In addition, this study was cross-sectional, and we could not conclude that there was a causal relationship between social participation and health condition. However, it was possible to describe differences in social participation between prefabricated housing and rented housing.

### Conclusions

Social participation was associated with the good health conditions of disaster evacuees. Residents in prefabricated housing had more frequent opportunities for social participation and seemed to obtain a larger health benefit from social participation compared to those who lived in rented housing.
